# Faculty as reflective practitioners in emergency online teaching: an autoethnography

**DOI:** 10.1186/s41239-021-00261-2

**Published:** 2021-05-17

**Authors:** Insung Jung, Sawa Omori, Walter P. Dawson, Tomiko Yamaguchi, Seunghun J. Lee

**Affiliations:** 1 Education and Language Education, College of Liberal Arts, International Christian University, 3-10-2 Osawa, Mitaka-shi, Tokyo, 181-8585 Japan; 2 Politics and International Studies, College of Liberal Arts, International Christian University, 3-10-2 Osawa, Mitaka-shi, Tokyo, 181-8585 Japan; 3 Asian Studies, College of Liberal Arts, International Christian University, 3-10-2 Osawa, Mitaka-shi, Tokyo, 181-8585 Japan; 4 Society, Culture and Media, College of Liberal Arts, International Christian University, 3-10-2 Osawa, Mitaka-shi, Tokyo, 181-8585 Japan; 5 Psychology and Linguistics, College of Liberal Arts, International Christian University, 3-10-2 Osawa, Mitaka-shi, Tokyo, 181-8585 Japan

**Keywords:** Autoethnography, Pandemic, Emergency online teaching, Faculty experience, Online education, Reflection-in-action, Reflective practitioner

## Abstract

This study aimed to chronicle and understand the emergency online teaching experience of five faculty members in a liberal arts college located in Tokyo, Japan during the COVID-19 pandemic of 2020, adopting the autoethnographic method. It explored the nature and dimensions of problems the faculty members faced, resources used to make sense of problems encountered, and actions they took to solve the problems as reflective practitioners in emergency online teaching. It also examined differences between faculty members over time. Analysis of seven weeks of autobiographic reflective journals during a 10-week academic term revealed that the faculty members encountered a range of problems during the classes, especially student-related and technology-related issues. When encountering problems, faculty members utilized references such as their past experience in face-to-face classroom teaching. Faculty members with more online teaching experience were more adaptable and flexible in mobilizing other references to solve problems. Overall, all members worked as reflective learners and practitioners who continued to reflect on the problems they faced and find solutions. These findings suggest that engaging in reflection-in-action and reflection-on-action can be effective ways for faculty members to develop their competencies to solve problems in emergency online teaching situations when responding to unprecedented challenges and issues is continuously needed.

## Introduction

The COVID-19 pandemic which started in December 2019 has created a major interruption to education at all levels across the globe. Bozkurt et al., (2020) estimate that over 1.5 billion students of all ages, nearly 90% of the global student population, have experienced a disruption in their education (UNESCO, 2020a; b). The International Association of Universities Global Survey was conducted in early 2020 with the participation of 424 higher education institutions from 109 countries. It revealed that classroom teaching has been replaced by distance education in 67% of the institutions and that more flexible learning possibilities, from blended or hybrid learning to emergency online teaching with synchronous and asynchronous platforms, have been utilized (Marinoni et al., 2020).

With the declaration of the state of emergency across all 47 prefectures in Japan from April to May of 2020, many Japanese universities, after a few weeks of delay for preparation, began to offer emergency online teaching. International Christian University (ICU), where this study took place, started the school year as scheduled with distance education and a brief preparation period of two weeks for emergency online teaching. When the shift to online education was first announced, faculty members who were generally not familiar with the use of online technology in teaching were in a panic and spent a great deal of time getting trained and preparing lectures. As they became more comfortable with the use of technology for emergency online teaching, they were able to handle technical and logistical issues with more confidence and began to pay attention to the pedagogical opportunities which online education presents.

This study aims to chronicle and understand the emergency online teaching experiences of five faculty members at ICU, a liberal arts college located in Tokyo, Japan during the COVID-19 pandemic. The five faculty members, who are also the researchers of this study, placed themselves in the role of participants and undertook a qualitative study wherein empirical data were derived from their autoethnography. Research questions included:

1. What were the nature and dimensions of problems (issues or doubts raised during online teaching), references (experiences or resources used to solve a problem) and actions (decisions or measures taken to solve the problem) of the faculty members during emergency online teaching?
2. How did the faculty members reflect on their actions and emergency online teaching in general?
3. What differences were observed in the faculty members’ experiences over time and between faculty members?

We hope that this study provides valuable insights into the ways by which individual faculty members responded to various challenges in emergency online teaching during the pandemic and adapted their techno-pedagogical approaches to a new teaching environment. We also hope that the study contributes to faculty development for online education even after the COVID-19 pandemic ends.

## Related work

When online instruction was first introduced in higher education in the mid-1990s, most faculty were reluctant to teach online (Olcott & Wright, 1995) and ill-prepared to make the shift from traditional classroom teaching to online course delivery due to a general lack of understanding of what it meant to teach online (Bates, 1999; Conceição, 2006; Lichtenberg, 2001; Palloff & Pratt, 2001) with little or no competencies to design and teach online courses (Harasim et al., 1998; Parker, 1997). For effective online instruction, faculty development, attitudinal and behavioral changes in teaching practices, and pedagogical strategies (Care & Scanlan, 2001; Harasim et al., 1998; Link & Scholtz, 2000; Palloff & Pratt, 2001; Watson, 2001) were highlighted in the literature.

With the rapid growth of online instruction, more studies have been conducted to explore faculty experiences in teaching online. Lao and Gonzales, (2005) investigated the attitudes, perceptions, and experiences of online teaching by faculty in a U.S. university and found that a faculty member’s personal preference for an online mode of teaching, level of technological expertise, and prior experience with online teaching were the important factors positively affecting their online teaching experience and the decision to teach online. Wingo et al., (2017), in a meta-study of 67 different research projects, concluded that “faculty were less satisfied with teaching online when they had technical problems” (pp. 21–22). Bolliger & Wasilik, (2009) similarly found that faculty satisfaction with online teaching is closely related to “using reliable technology and experiencing difficulties with technology” (p. 113). Nevertheless, instructors with more experience were generally more satisfied with online teaching (Wingo et al., 2017), showed a favorable attitude toward online teaching (Ulmer et al., 2007) and had higher perceived self-efficacy (Lao and Gonzales, 2005). Conceição (2006) offers a typical summation of the online teaching experience with the following quote, “Teaching online is a lot of work, but it is also rewarding" (p. 40). Similarly, De Gagne and Walters (2009) revealed that faculty members perceive online teaching as a flexible, convenient and learner-centered mode of teaching even though it is time-consuming and labor-intensive and needs strong institutional support and continuous training.

Similar experiences were reported in a few studies that investigated faculty experiences with online course delivery during crises such as natural disasters or socio-political turmoil. Fox, (2007) interviewed eight teachers to describe their experiences and to share stories of success and failure in the use of Information and Communications Technology (ICT) during school closure due to the Severe Acute Respiratory Syndrome (SARS) crisis in 2003 in Hong Kong. That study revealed that teachers were able to see both the potential and limitations of ICT in instruction and that the negative effects of ICT were mostly due to lack of preparation time and teacher competence. Lorenzo, (2008) reported faculty experiences of online teaching in the Sloan Semester project implemented at over 100 higher education institutions after Hurricane Katrina and revealed faculty members’ general sentiment that academia is often slow to adopt new tools and innovations. Tull et al., (2017), after analyzing findings from five studies investigating the practices of a university during the New Zealand earthquakes (2010–2011), identified the importance of communication and professional development for faculty in promoting the adoption of e-learning and fostering resilience for faculty to shift to online delivery during crises. Tauson and Stannard, (2018) who reviewed 135 publications for effective technology use during the Syrian conflict also pointed out the importance of professional development and communication for teachers. Czerniewicz et al., (2019) investigated attitudes of academics who taught blended and online learning courses during student protests in South Africa between 2015 and 2016 and revealed that most members accepted both blended and online approaches as viable options during the shutdowns. These studies demonstrate that faculty come to understand both the potential and weaknesses of online course delivery during crises and that they need technical support, communication, and capacity building for effective blended or emergency online teaching.

A small number of studies investigating faculty experiences with emergency online education during the Covid-19 pandemic have been published so far. Johnson et al., (2020) conducted a survey investigating experiences of faculty and administrative staff from 672 institutions in the USA in the early weeks of emergency online teaching. It was revealed that faculty with and without prior experience were able to adopt new teaching approaches and tools and made adjustments in the assignments, exams and grading policies accordingly and recommended the provision of online professional development opportunities for faculty. Shenoy et al., (2020) interviewed 20 faculty from Indian universities and found that some faculty felt that online technology was a disturbance at first, but then quickly developed a habit of adopting virtual modes of teaching and began to perceive technology as an enabler for pedagogical innovation. Similarly, a survey conducted with 59 faculty members in Saudi Arabia (Almaghaslah & Alsyari, 2020) revealed that most participants agreed with the smooth shift to emergency online teaching and liked its flexibility. However, they also noted the need for faculty support and more active utilization of online technology for research, community service, and conference participation. Lack of faculty’s online teaching experience, early preparation, and institutional support were also reported in Peking University in China where over 4400 courses were hurriedly offered online (Bao, 2020). These studies on faculty experiences during the early stages of the COVID-19 pandemic are instructive as they reveal faculty members’ confusion, anxiety, and struggles and thus offer direction for subsequent stages. However, as these studies collected data at the early stage of the shift to emergency online teaching, and within a short period of time, they could not provide a comprehensive view of faculty reflection on their experiences with emergency online teaching and changes which have occurred after the introductory stage.

Donald Schön’s reflection-in-action framework can be applied as a means to reveal what faculty face, think, and do in their online teaching practice. As Schön, (1983) implied in his framework, it is assumed that faculty members who have been teaching online are reflective practitioners in this new environment, having a continuous conversation with the emergency situation and themselves. The reflection-in-action framework explains how a professional (e.g., an instructor) does an action (e.g., teaching with a certain pedagogical approach), that action generates an effect (e.g., student learning), and this effect serves as feedback for the professional to repeat, reframe or modify her/his approach (e.g., the pedagogical approach) and continue her/his “conversation with the situation” through a new action (e.g., teaching with a revised new pedagogical approach).

This study adapted Schön’s reflective-in-action framework for the research purpose and applied three categories of the reflection cycle in the initial coding stage: 1) “descriptive reflection” which involves setting and identifying the problem (coded as *problem*), 2) “comparative reflection” which includes thinking about the matter for reflection in consideration of different perspectives or references (coded as *reference*), and 3) “critical reflection” which describes thinking about possible solutions or actions considering the different perspectives or references (coded as *action*) (Jay & Johnson, 2002, pp. 77–79).

## Methodology

The present study was conducted in order to collect autoethnographic data from five ICU faculty members during a 10-week spring term in 2020. ICU had integrated Moodle and other digital tools in its in-person teaching previously but never offered fully online courses prior to the COVID-19 crisis. The researchers embarked on a qualitative study consisting of autoethnographies of themselves as research participants in the study to generate empirical data.

Autoethnography is defined as a form of personal narrative that explores the author’s lived experiences (Mallet, 2011) and is considered “an approach to research and writing that seeks to describe and systematically analyse (graphy) personal experience (auto) in order to understand cultural experience (ethno)” (Ellis et al., 2011, p. 1).

### Auto: researchers as participants

The participants in this study were the five faculty members who taught emergency online teaching during the COVID-19 pandemic in Spring Term 2020 (Table 1). This group of faculty members with varying levels of prior exposure to online teaching was chosen to represent a sample of faculty according to gender, age, and academic discipline to examine emergency online teaching experiences from diverse perspectives. Although convenience sampling was used in selecting participants, what was common among them was their strong interest in reflecting on and analyzing their teaching experiences during one 10-week term of emergency online teaching.

**Table 1:** Research participants

Participant ID	Gender	Experience	Specialization
F1	F	Taught in S. Korea, the USA and Japan for 30 yrs. Extensive experience with various technologies and online teaching	Educational technology
F2	F	Taught in Japan for 12 yrs. Used basic functions of Moodle. No previous experience with online teaching	International political economy & public policy
F3	F	Taught in Japan for 14 years. Used basic functions of Moodle. No previous experience with online teaching	Sociology
M1	M	Taught in S. Korea, the USA and Japan for 21 years. Used Moodle, Blackboard, Sakai extensively. Some experience with online teaching	Linguistics
M2	M	Taught in the USA, S. Korea and Japan for 18 years. Used Blackboard, Google Suite, and Moodle extensively. No previous experience with online teaching	Asian Studies

### Ethno: research contexts

We, as the researchers of this study, kept a journal regarding our experiences and observations in emergency online teaching for seven weeks during the spring term (between April 9 and June 17, 2020). In total, we recorded 35 journal entries. Each of us chose one online course (Table 2) and reflected on our teaching experiences, thoughts, and feelings in each journal entry.

**Table 2:** Five online courses selected for journal writing

Course area (Level)	Instructor	Enrollment	Course structure and technology
Technology Application (Introductory)	F1	40	Zoom and Moodle as main platforms. Kahoot, Google drive & Mentimeter as supportive tools. A combination of lectures, discussions and student presentations
Research Method (Advanced)	F2	27	Zoom and Moodle as main platforms. Workshops, discussions and student presentations with no lecture component
Social Issues (Graduate seminar)	F3	5	Zoom and Moodle as main platforms. A combination of lectures, discussions and student presentations
Linguistics (Introductory)	M1	35	Webex, Slack, and Moodle as main platforms. Mentimeter as supportive tool. A combination of lectures, and discussions
Education (Intermediate)	M2	29	Pre-recorded lectures uploaded as unlisted YouTube videos combined with weekly discussion on Zoom. All course materials managed on Moodle. Some experimentation with Mentimeter

### Graphy: journal writing and analysis

Prior to the start of data collection, approval from the Research Ethics Committee of the researchers’ institution (#2020–10) was obtained. No personal information of the students, teaching assistants or co-instructors was included in any of the journal entries.

In the weekly entry, each of us described the context of our online classes (date/, period, tools used, teaching methods, etc.), addressed the following questions and added more reflections on our teaching:

- Overall experience: What were your main teaching behaviors or activities? What were you thinking and feeling while you were teaching?
- Reflection: Did you face any expected and/or unexpected problems or issues? Did you address/solve some or all those issues? What did you think about those solutions? Were any knowledge/skills from your prior classroom teaching experience useful? Were there any other knowledge/resources that were helpful? Once the class was over, what did you think and feel about your class? Do you have any reflections or appraisals on the problems or issues solved and unsolved?

Upon completion of journal writing, we carefully read all journal entries to familiarize ourselves with the data and develop a big picture of our experiences and reflections during emergency online teaching. After sharing our understanding of the data, we developed the initial coding scheme consisting of three themes (problem, reference, and action) that were adapted from Schön`s reflection-in-action framework of descriptive, comparative and critical reflection and one additional theme (self-appraisal). We then analyzed the data using MAXQDA (v20.2.1) following three phases.

- Deductive coding phase: Each of us carried out the analysis of our own journal data applying the four themes listed above and explored sub-themes for the next coding phase.
- Inductive coding phase: We generated sub-coding schemes. The unit of analysis was a phrase with a single idea. After careful review of the coded data, we created tentative labels for data chunks, grouped them as sub-themes which were repeatedly modified as a group.
- Final cross-checking phase: Individual coding was cross-examined by other members. Variations in our coding were discussed and resolved in a series of bi-weekly discussions of key themes and sub-themes for two months.

### Findings

#### Faculty experience and reflection of emergency online teaching

To address the first and second research questions, we used four themes as the initial coding scheme: problem (an issue raised during online teaching), reference (an experience or a resource used to solve the problem), action (a decision or measure to solve the problem) and self-appraisal (reflection or assessment during the class or the whole teaching situation). Table 3 reveals sub-themes which emerged in each theme category.

**Table 3:** Coding and categorization of faculty experiences during emergency online teaching

Theme	Subtheme	Code frequency (%)
Problem	Student-related	72 (25.4)
	Technology-related	68 (23.9)
	Content-related	43 (15.1)
	Time-related	41 (14.4)
	Adaptability	28 (9.9)
	Logistical	12 (4.2)
	Confidence	11 (3.9)
	Others	9 (3.2)
	Sub Total	284 (100)
Reference	Classroom teaching	60 (49.2)
	Students	18 (14.8)
	Previous experience with online teaching	17 (13.9)
	Peer	11 (9.0)
	Teaching assistant (TA)	5 (4.1)
	Training	4 (3.3)
	Research evidence	4 (3.3)
	Knowledge of technology	3 (2.5)
	Sub Total	122 (100)
Action	Pedagogical solution	78 (37.9)
	Technical solution	48 (23.3)
	Logistical solution	44 (21.4)
	Getting support	18 (8.7)
	No action	13 (6.3)
	Emotional adjustment	5 (2.4)
	Sub Total	206 (100)
Self-appraisal	Feeling positive	114 (37.6)
	Identifying prospects	79 (26.1)
	Feeling negative	67 (22.1)
	Regret	22 (7.3)
	Mixed feelings	20 (6.6)
	Seeking empathy	1 (0.3)
	Sub Total	303 (100)

We, as faculty members, faced a variety of problems during emergency online teaching; however, as shown below, student-related and technology-related problems were most common across all members.

- Student-related problems (25.4%) included problems ranging from students showing lack of motivation to students having difficulties in adjusting to self-directed online learning. F1 stated, “*One thing I noticed was that most students who appeared on the screen did not show any emotions on their face*.” This could be lack of motivation or difficulties in adjusting on the part of students which is difficult to discern online. On the other hand, F3 noted positive changes in students’ response to online learning over time, which could be an indication that students are motivated. However, F3 observed that the *“differences among students in terms of their motivation and ability have become clearer.*”
- Technology-related problems (23.9%) included difficulties faculty faced in using hardware or software for online teaching. Zoom-related problems using breakout rooms were most common for synchronous teaching.
- Content-related problems (15.1%) included concerns over the level of difficulty of reading materials and the degree of students’ understanding in the classes. Students had difficulty finding academic papers online in Japanese and thus read more academic papers in English which F3 perceived to be a positive learning experience for them; nevertheless, she also expressed the feeling that, “*I ought to provide support on this*.”
- Time-management problems (14.4%) included challenges such as time constraints after facing unexpected technical problems and management of time to allow for questions or discussion.

When faced with different types of problems, we sought various references that included our previous in-class teaching experiences, student suggestions and our previous online teaching experiences.

- How we taught the same course in the classroom was the most prevalent reference among all other options (49.2%). For instance, compared with online teaching, M1 found that “*explaining the examples in an in-class setting would have been easier because I would have seen the reaction by the students*.” On a similar note, F3 anticipated challenges in teaching an action-oriented course online in a future term and stated, “*I would very much like to hear experience of other people who have taught those types of classes*.”
- Students (14.8%) provided advice to their teacher when the teacher faced a problem. When F2 was not able to find the breakout function button with her guest status, a student explained how to solve that problem.
- With respect to previous online teaching experiences, M2 recalled that “*in the previous class we divided the students into their debate teams to practice using the manual assignment function of breakout rooms which worked OK*.”

Interestingly, formal and informal training materials (3.3%) and related research evidence (3.3%) were not used much as tools to solve problems in emergency online teaching.

Actions taken to solve the problems during emergency online teaching were mostly related to the application of pedagogical strategies (37.9%), technical solutions (23.3%), and logistical arrangements (21.4%).

- Pedagogical actions were extensively adopted to address a wide range of problems even for technological and logistical issues. M1 used online comment sheets “*to grasp what students understood and what they have not yet clearly grasped*” and F3 adjusted the contents of lectures “*based on the predictions of what students would be able to understand and what would not*” when they could not look at students’ reactions on the Zoom screen. When F2 observed that students were absent because of internet connectivity issues, F2 made sure that those students would not lag behind in learning by sending out announcements via Moodle.
- Logistical actions addressed mainly logistical issues such as language of instruction and class preparation, and technical actions addressed problems mainly related to newly adopted technologies (e.g., Zoom and Kahoot).

In appraising online teaching in general and our problem-solving behaviors, we felt either positive (37.6%) or negative (22.1%), and identified prospects for future actions (26.1%). In a few cases, we expressed regret (7.3%) or mixed feelings (6.6%). F2 worried that “*sometimes I had to give tough instructions for students to let them reorganize the construction of their research papers, so I had concerns over whether my comments toward students were too severe*.” M2 experienced a case where he wanted to view statistics on a website with students during a Zoom class, but the website was not displaying properly. The experience left him with negative feelings but also prompted him to plan what future actions he would take to overcome technical difficulties.

#### Differences and similarities in faculty experience and changes over time

To answer the last question, we analyzed the differences and similarities in emergency online teaching experiences among us and changes in problems, references, actions and reflections over time.

In the *problem* category, we found no noticeable differences among us. But technical problems that each of us faced varied depending on technology type (synchronous vs. asynchronous), tools and materials used, and pedagogies adopted during online teaching.

- Those who implemented Zoom often faced technical issues in implementing small group discussions. F1 and M2 had a problem in assigning students into small groups manually using Zoom's breakout rooms function.
- F3 who used YouTube video clips faced another issue. She could share a video but without sound.
- M1 who introduced another interactive platform, mentimeter, during his Zoom sessions stated that its "*anonymous comment sheets …prevented me from knowing whether the questions were coming from many students or whether only a handful of students were asking questions*."
- Those who introduced asynchronous sessions had different problems. M2 faced an issue in recording a lecture using Zoom: "*Although I was clicking from slide to slide while delivering the lecture, in the Zoom video the PowerPoint stayed on the first title slide. So, in effect, I had just displayed the title slide for 55 min!*"

We identified no noticeable changes in the problems over time except that problems related to adaptability to online teaching declined as we became more adapted to online teaching and technologies.

In the *reference* category, we noted both similarities and differences among us, especially depending on the years of experience with online teaching and technology.

- Both groups, those who had more experience (F1 and M1) and those with less experience (F2, F3 and M2) with online teaching, used classroom teaching as their predominant reference point in the problem-solving process and listened to advice from students. M1 stated that “*explaining the examples in an in-class setting would have been easier because I would have seen the reaction by the students*.” F2 also claimed that in classroom teaching, "*I could more easily oversee which group had difficulty and which group went well during discussion.”*
- Those with more online teaching experience tend to use references beyond classroom teaching. Research evidence, advice from their colleagues and teaching assistants, and previous experiences with online teaching were sought to understand the problems and explore possible solutions. F1 stated, "*I looked for some papers and found a few useful references. Even though they did not have an exact answer for my specific question, one article was helpful*." M1 and F1 also relied on TAs to monitor students' responses. On the other hand, those with less experience with online teaching used classroom teaching as their main reference when faced with various problems.

Those with less or no online teaching experience got accustomed to online teaching as time passed and were able to handle the problems by referring to their own online teaching experiences from previous weeks in the same course as references. For instance, when encountering a problem in allocating students by Zoom's breakout functions in the mid-term, M2 stated that "*usually this was solved by re-sending the invitation a couple of times*” based on his experience in the early stage of online teaching.

In the *action* category, we found more similarities than differences among us.

- We all had to respond without much delay to the problems, especially technology-related ones, that suddenly disrupted the class. M2 was quick to respond to a situation when his camera was off by resolving the connectivity problem. F2 also responded to the students’ connectivity issue as soon as it happened by directing students “*to turn off the PC and try to log out from the browser entirely and then sign in using the university account*."
- We all proactively took both pedagogical and technological actions to improve the quality of online teaching. M2 added a new interactive tool, mentimeter, during his Zoom session and stated that, *"After the lecture I shared the students’ 9 questions which were uploaded to menti.com by sharing my screen. I felt like this was more questions than would have been asked in class…*” F1 integrated Google functions during the Zoom meeting and posted detailed instructions and a link to the Google doc. She noted, “*I did not have to visit each breakout room, which often disturbed the discussion. Instead, I read what each group was typing and left my comments in the Google doc*.”

As time passed, we tried out new pedagogical, technical, and logistical solutions. M1, who used to write on the whiteboard to explain complex concepts during classroom teaching, wrote that “*I write notes on the pdf that students can look at … I found this method to be useful for delivering complex concepts that are not immediately understandable*.” Then, over time, directly annotating the PDF “*became a standard way of making notes when explaining concepts*” for M1. F2 utilized the screen-sharing function of the Zoom technology and made all students share their in-progress research papers in small groups and offer peer review on each other’s work. F3, who used to provide advanced students with additional materials and less advanced students with consultation during her classroom setting, utilized the Moodle function together with Zoom to offer similar support. She stated that: “*I uploaded additional materials and slides to help students prepare assignments in addition to class materials and discussions. I also made appointments with students who tended to be less vocal in the online class*.”

In the *self-appraisal* category, we observed interesting differences between those with more online teaching experience and those with less experience even though we all had both positive and negative feelings and discussed the prospects for future online teaching.

- Around 28% of self-appraisal codes of those with less online teaching experience expressed negative feelings, while about 14% of the codes of those with more experience stated negative feelings. That is, M2, F2 and F3 who had less online teaching experience assessed online teaching more negatively compared with F1 and M1 who had more experience. M2 expressed his frustration and had to remind himself that, "*I had become too trustworthy of the technology in thinking that it would work reliably each and every time*." F2 also expressed frustration that, “*the differences among students concerning their motivation and class engagement are visible from the beginning of the term …and such differences seem to persist than classroom teaching*.”
- Unlike M2 and F2, the negative feelings of F1 and M1 were less toward online teaching environments or technology functions, but more toward class content and lack of discussion.

As time passed, we became more confident and competent and felt more positive with online teaching. F1 stated that "*I found myself looking around students’ faces on the screens and trying to read their reactions to the video, which I could not do calmly a few weeks ago*.” Also, our negative feelings decreased while positive feelings increased, and we began to talk more about prospects for future classes. In the first week of the class, M2 said, “*Honestly, I was kind of fed up with the Zoom software. It is infuriating when a product does not work as it should. I don’t like the feeling that students might not trust me to be doing the right thing when it is because of some faulty product*.” Then toward the end of the class, he felt positive when he was able to overcome the technical problems. M1 who said, “*it is quite tiring because many things need to be managed (more than a traditional class) at the same time*” in the first and second weeks of his class, even felt happier later in the term noting that, “*I was happy that there were all these questions because it would have been difficult for me to know the degree of confusion or knowledge*" after a long online Q&A session. Despite initial negative assessment, F2 also stated that online teaching could “enhance the mutual learning among students than I expected,” which recognized the benefits of online teaching in the last week. F1 expressed her preference for a combination of online and classroom teaching for the future, emphasizing the necessity of online teaching skills in writing, “I prefer teaching classes blending face-to-face and online approaches…my full understanding of potentials or affordances of various technologies is important for an active learning process and better outcomes.”

## Discussion

Similar to those who taught online courses during non-crisis situations, we also found technical problems (23.9%) to be a serious issue which created negative feelings toward online teaching. As our level of technological competence and experience with online teaching improved, those feelings decreased and positive feelings increased as found in the previous studies conducted in non-crisis online learning situations (e.g., Lao & Gonzales, 2005; Wingo et al., 2017). While technical problems can happen unexpectedly in any online course, contingency plans need to be developed and students need to be notified in advance, especially in the early stages of a period of emergency online education when faculty are less familiar and experienced with online tools (Bao, 2020). For example, in a Zoom class, another synchronous meeting room (e.g., Google Meet) can be used as a contingency plan.

It was surprising that, compared with online learning in normal situations (e.g., Goh et al., 2017), we noted more issues related to students’ understanding of the content and materials, their reactions to the lecture, and their psychological and physical well-being (25.4%). This may be explained by the fact that we had little time and competence to foresee those pedagogical issues in advance because this was a case of emergency online education and thus, we naturally became more sensitive to the student-related issues and received more requests from the students asking for help with online learning. In fact, all of us reported two main problems that our students often faced in online instruction: difficulties in understanding the lecture content and/or discussion topics due to technical or time constraints (F2, F3 and M2) and a low level of attention and participation (F1 and M1). To address these problems, we often sought solutions from our in-person classroom teaching, even though the previous literature indicates that face-to-face teaching is not always a suitable reference for designing a successful online course.

As summarized by Baran et al., (2013), while teachers’ traditional roles in classroom settings can be transferred to online instruction, the affordances and limitations of the online environment require teachers to play new roles and acquire new competencies to help students achieve successful and meaningful learning outcomes. To adapt to the new roles, several studies offer effective strategies specifically for online teaching including: offering clarity and a clear structure of the learning content (Lee & Lee, 2008), dividing the learning content into smaller chunks (Bao, 2020), promoting structured communication and high teaching presence (Orcutt & Dringus, 2017), and providing support and encouragement especially for quiet students (Paechter & Maier, 2010). To practice these strategies successfully for online teaching, it is argued that online teachers need to develop some new competencies that have not been emphasized in classroom teaching situations, including but not limited to: facilitation and management skills (Palloff & Pratt, 2001), pedagogical competencies including capabilities to define online participation, communication of course goals and outcomes, tracking of students’ learning process and performance (Bawane & Spector, 2009; Berge, 2009; Bigatel et al., 2012), skills to access and use various technological resources and tools (Albrahim, 2020; Egan & Akdere, 2005), skills to design online participation and assessment (Palloff & Pratt, 2001), and competencies to promote social presence (Albrahim, 2020). Among these competencies, our research reveals that technology-related competencies and pedagogical competencies are more important for faculty teaching online during a crisis, compared with competencies related to facilitation and communication, management, assessment, and affective and social support (Berge, 2009; Coppola et al., 2002) which would become important at the later stages of emergency online teaching.

Our research confirmed the results of previous studies indicating that faculty members’ level of prior experience with technology use in instruction, among other factors, affects the way faculty utilize resources to solve their problems during online teaching. However, our research added a new finding regarding how faculty members’ level of online teaching expertise affected their decision to utilize resources to solve the problems. The three faculty members with less online teaching experience applied strategies mostly from their classroom teaching in addressing various issues raised during emergency online classes, while the members with higher levels of online teaching experience utilized not only classroom teaching experiences but also other sources such as findings from previous research on online learning and advice from skilled assistants and their colleagues. This finding can be explained in relation to well-established literature on differences in teaching practice and decision-making between novice and experienced teachers. Compared with novice teachers, experienced teachers are known to have a greater amount of content and pedagogical knowledge and continue to structure and scaffold such knowledge around problems and cases encountered in teaching (Lachner et al., 2016), adapt their teaching to meet the needs of students rather than just follow pre-planned objectives (Westerman, 1991), develop expertise in a certain domain through practice, use previous experiences and reflection (Raduan and Na, 2020), and be more optimistic, flexible and insightful in their teaching (Berliner, 2001). Similarly, experienced online teachers tend to adapt themselves to the online teaching environment more resiliently and readily compared with less experienced teachers, by building on their previous experiences, continuously engaging in pedagogical inquiry processes with themselves and their students, and constantly reflecting on their assumptions and perspectives on online education (Baran et al., 2013). In our study, two faculty members who had prior experience with online teaching showed several features of experienced teachers such as being more adaptable and flexible in mobilizing resources to solve problems than their counterparts with less experience at the early stages of emergency online learning. However, as the faculty members with less experience developed expertise in online teaching through experience and reflection over a relatively short period of time, they became more aware of students’ problems and needs, began to explore more resources and options suitable for online teaching when faced with problems, and became more flexible and optimistic, as argued in Berliner (2001). Further investigation into consequences of those options and solutions adopted by faculty with different levels of online teaching experience on students’ satisfaction and learning outcomes would help us understand the effects of different technological, pedagogical and logistical strategies in emergency online teaching.

**Fig. 1:**
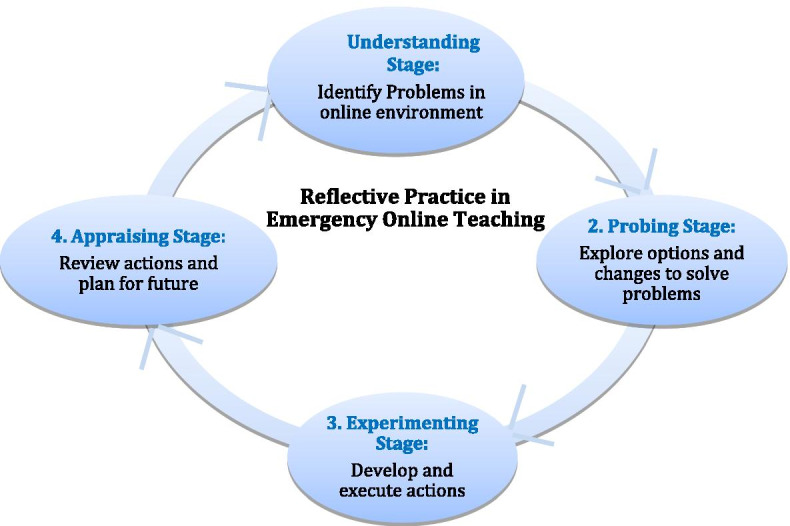
Reflective practice cycle during emergency online teaching

On the whole, we worked as reflective practitioners who continued to reflect on the problems we faced during online teaching and carry out reflection-in-action involving “thinking on (our) feet” as Schön (1983, p. 61) described. Moreover, we tried to solve problems by referencing options from our past classroom or online teaching experiences, which could be described as reflection-on-action involving “thinking back events” (Schön, 1983, p. 61). All these findings support Schön’s (1983) argument that both reflection-in-action and reflection-on-action are at the core of professional development and new learning when responding to uncertainties is essential. Regardless of our level of technology expertise we felt frustrated and stressed from emergency online teaching conditions and technical challenges especially at the early stage of the period of emergency online teaching. Nevertheless, we became active learners who eventually acquired the necessary skills to overcome technical problems even under dire circumstances. We also became optimistic educators who used our professional knowledge from classroom teaching for the purpose of providing better opportunities for students' active learning in emergency online education and improving our teaching in the future. Osterman and Kottkamp (1993) explained this reflective practice as “a means by which practitioners can develop a greater self-awareness about the nature and impact of their performance; an awareness that creates opportunities for professional growth and development” (p. 2). As identified in Schön (1983), Manti et al., (2011) and Russel (2005), we executed reflective practice across several stages. First, we developed our understandings of the situation (understanding stage), explored references and changes in our approach to solving problems (probing stage), developed and executed new courses of action (experimenting stage), and reviewed our own actions and came up with the ideas developed as a result of this review (appraising stage). This reflective practice cycle (Fig. 1) can be applied in training and supporting faculty members to reflect on and continuously improve their online teaching during and after the COVID-19 pandemic.

## Conclusion

Adopting an autoethnographic approach, we chronicled and analyzed our attitudes and actions during a 10-week academic term of emergency online teaching as reflective practitioners. We found that teaching online without much preparation is a challenging experience. We learned that engaging in reflection-in-action and reflection-on-action can be effective ways for us to develop our competencies to solve problems in emergency online teaching situations when responding to unprecedented challenges and issues is continuously needed. We hope that the reflective practice cycle during emergency online teaching proposed in this study (Fig. 1) can be applied to evaluate the effectiveness of proposed strategies in future empirical studies and improve practices in online higher education. While our findings result from a special type of “emergency online teaching” which made preparation time limited, we feel that our findings may also be relevant for non-emergency situations where the impetus for starting online education comes from external motivations, such as providing education during a pandemic, rather than from the will of the instructor. In this sense, it is imperative to understand the challenges that are faced by instructors with limited previous online teaching experience as well as strategies to assist them.

One limitation of this study is that the results obtained from the autoethnography of the five researchers who taught relatively smaller classes in a liberal arts college in Japan may not always be transferable to other contexts. Future research engaging more participants who teach online classes of different sizes and in various teaching situations for a period longer than a 10-week academic term is recommended. Although the reflective practice cycle is proposed based on the findings from our autoethnographic data, its phases and components may require further evaluation and validation in diverse contexts and with quantitative data.

## Data Availability

The datasets used and/or analyzed during the current study are available from the corresponding author on reasonable request.
